# The Hygiene of Motherhood

**Published:** 1890-11

**Authors:** John Sheppman


### HALL'S JOURNAL OF HEALTH

TRUTH DEMANDS NO SACRIFICE; ERROR CAN MAKE NONE.

Vol. 37. NOVEMBER, 1890. No. 11.

### THE HYGIENE OF MOTHERHOOD.

By Dr. John Sheppman.

PART FIRST.

There are few young women who thoroughly understand the responsibilities which they take upon themselves when entering into the holy bonds of matrimony. Mothers, as a rule, do not impress upon the minds of their daughters the importance of hygienic precautions, which are absolutely necessary for the maintenance of health and happiness in every household. The first duty and foremost ambition of every woman ought to be to know how to cook well, and to make a practical study of what we ought to eat, and what we ought *not* to eat.

The art of cooking is one in which every industrious person can easily become experienced, and, with a little patience, can gain an unlimited knowledge of its various branches. There is an old adage which tells us that "man grows like what he feeds on," and if this is in the slightest degree true, it certainly should be a warning for us to cut down the quantity of pork, beef and all other animal foods, the consumption of which is not at all necessary for the existence of man, and which have been frequently discovered to be the prime factors in the propagation of disease.

Pork, as an article of food, should be condemned by every medical practitioner, and placed upon the prohibitory list of every sanitarian. From earliest history we learn that the flesh of the hog was abhorred by the Hindoos, the Ethiopians, the Phœnicians, the Egyptians, the Scythians, and possibly many others. The Talmud or Hebrew laws declare that "ten measures of pestilential sickness were spread over the earth and nine of them fell to the share of the pigs." Mahommed cautioned his people against the use of it, and Moses, with his wealth of spiritual wisdom, was not slow to denounce its use throughout all Israel. The Pythagorians were taught to abstain from eating all meats, and the Bedouins declared the hog to be the only animal whose presence and touch were stamped with pollution. Statistics can be shown to prove that the death rate among foreign soldiers from scrofulous diseases is about twenty-five per cent., and as pork enters largely into the diet of those military subjects, I feel no hesitancy in siding with a recent declaration that "scrofula and tuberculosis are diseases directly communicated from hog to man." It must, therefore, be very apparent that the eating of any portion of this animal's flesh is not only unhealthy, but positively dangerous. I cannot understand how any person can continue to relish pork after seeing, or even reading about the filthy, loathsome and swiltering habits of the pigs.

The muscle parasite, or Trichina-spiralis has been discovered in the flesh of seemingly healthy hogs, and, when taken into the stomach, cause the most intense suffering, which is nearly always followed by a painful, lingering death. Experiments upon this subject have proven that about 170,000 of these interesting animals can live and propagate upon a square inch of the human muscle. There are various methods employed to detect its presence. The best means, however, is to submit a piece of the diseased meat to a light solution of liquid potassa for about thirty minutes, after which, by the aid of a microscope, they can be plainly observed.

It was not my purpose to make this article a lengthy dissertation upon the hog, its flesh, or its pedigree ; but I certainly do hope that I

have sufficiently delineated the facts as they are presented to me, for the exercising of an alterable influence upon all those who have hitherto been daily partakers of this forbidden food.

There are no fixed or distinctive laws governing the diet of all people ; for what disagrees with one, does not always prove likewise with another. Be this as it may, there are two well known conditions of food which have been scientifically proven to be detrimental to the health of every one, and they are—food that is not sufficiently cooked, and all "fried abominations." The question may be asked, "What should we eat if all meats are to be cut from our dietary regimen ?" In answering this, permit me to say that there is no part of the human organism that cannot find its subsisting nutriment in fruits and vegetables. The ox, the cow, and the horse, are fed upon corn, oats and hay, and no one will question whether they gain physical and muscular development upon these substances. If we study the phylogenesis of the human race, we will learn that longevity was a notable feature among the early Hebrews, and that the custom of meat eating had not at that time been established. Nature never intended the lower animals to be slain and sliced for food. Scientists have urged against it ; and physiologists may some day discover the true earthly mission which an all wise Providence has set apart for them.

"For nought so vile that on this earth doth live,
But to the earth some special good doth give."

I do not wish my readers to look upon me as a staunch vegetarian, nor one who is thoroughly bent upon ruining the butcher and his honorable craft. But I certainly do think that it would be considerably better for all classes of humanity if every one would be a little more economical in regard to the consumption of all animal foods. If you *must* have flesh, by all means exclude pork. And whatever your selection may be, endeavor to have it broiled, cooked or roasted. Do not allow your meat to lay or hang in a room where there are tobacco fumes. Recent experiments by a Parisian inspector have proven that a small quantity of minced beef, held to the fumes of tobacco, had absorbed enough poison to cause the death of a dog in thirty minutes. This does not only apply to raw meats, but to those that are prepared for the table as well.

The superiority of man, over beast, does not confirm itself in the relative fitness to select or reject articles which are not congenial to the intricate movements of the stomach. Unfortunately our palates are artificially seasoned, susceptible beyond control, and the craving, unnatural appetite is permitted to rule our senses to such an extent that we invariably find ourselves victims of the injudicious habit of "gormandizing."

The influence of diet upon the character of people is a subject of grave importance, and one upon which we should devote our future studies. Some time ago, while seated in a court of justice, I noticed a poor, delicate, half starved young woman, appear against a red faced, burly, savage looking fellow, who was charged with the lamentable crime of wife beating. The Judge, in pursuance of his duty, asked: "How long have you been married?" "Four months," came the reply. "Are you a drinking man?" "No, sir." "What are your reasons for abusing your wife?" "I can't answer that, Judge; I suppose it is my pugnacious disposition." "Is she careless or does she neglect her household duties?" "No, sir. She's a model little woman in every respect. It's all my fault, Judge, but I can't help it." Turning to the wife, the Court asked: "What have you to say against him?" "Nothing," she sobbingly answered, "but that I will forgive him if he promises to behave himself in the future." "What do you feed him on, raw meats?" "No, sir," was the feeble response. "How often do you give him meat?" A little reflection elicited the fact that he was served with meat three times a day and occasionally for lunch. "Take my advice," said the Judge, "and cut off his flesh supply. The animal passion and hankering thirst for blood can only be subdued in this way. Take him home, he is full of remorse, and if he appears before me again, I'll send him to the work-house, and order him to be fed upon stale bread and vegetables."

I felt sufficient interest in this case to study it up, and to discover that the poor little abused wife did actually follow the prescribed course, and that her husband was not slow to regain his human instincts, and to awaken to a knowledge of the fact that he, indeed, was one "whose brightest joy was that he called her—'wife.'"

